# The influence of cytotoxic drugs on the immunophenotype of blast cells in paediatric B precursor acute lymphoblastic leukaemia

**DOI:** 10.2478/raon-2024-0006

**Published:** 2024-02-21

**Authors:** Tomaz Prelog, Simon Bucek, Andreja Brozic, Jakob Peterlin, Marko Kavcic, Masa Omerzel, Bostjan Markelc, Tanja Jesenko, Veronika Kloboves Prevodnik

**Affiliations:** Department of Haemato-Oncology, University Children’s Hospital, University Medical Centre Ljubljana, Ljubljana, Slovenia; Faculty of Medicine, University of Ljubljana, Ljubljana, Slovenia; Department of Cytopathology, Institute of Oncology Ljubljana, Ljubljana, Slovenia; Institute for Biostatistics and Medical Informatics, University of Ljubljana, Ljubljana, Slovenia; Department of Experimental Oncology, Institute of Oncology Ljubljana, Ljubljana, Slovenia; Institute of Pathology, Faculty of Medicine University of Maribor, Maribor, Slovenia

**Keywords:** immunophenotypic changes, chemotherapy, immunotherapy, paediatric B-ALL

## Abstract

**Background:**

Flow cytometry plays is important in the diagnosis of acute lymphoblastic leukaemia (ALL) and when antigen-specific immunotherapy is indicated. We have investigated the effects of prednisolone, vincristine, daunorubicin, asparaginase and methotrexate on the antigen expression on blast cells that could influence the planning of antigen-specific therapy as well as risk-based treatment assignment.

**Patients and methods:**

Patients aged ≤ 17 years with *de novo* B-cell ALL (B-ALL) were enrolled in the study. Blast cells were isolated and exposed *in vitro* to 5 individual cytotoxic drugs in logarithmically increasing concentrations. Then, the expression of CD10, CD19, CD20, CD27, CD34, CD45, CD58, CD66c and CD137 antigens was determined by quantitative flow cytometry.

**Results:**

Cytotoxic drugs caused dose-dependent or dose-independent modulation of antigen expression. Daunorubicin caused a dose-dependent down-modulation of CD10, CD19, CD34, CD45 and CD58 and an up-modulation of CD137. Vincristine caused a dose-dependent down-modulation of CD19 and CD58 and an up-modulation of CD45. Daunorubicin also caused dose-independent down-modulation of CD27 and prednisolone down-modulation of CD10, CD19, CD27, CD34 and CD58. Down-modulation of CD20 was detected only in relation to the specific dose of daunorubicin.

**Conclusions:**

The results of the study have shown that cytotoxic drugs can alter the expression of antigens that are important for immunotherapy. Importantly, daunorubicin, prednisolone and vincristine caused down-modulation of CD19 and CD58, suggesting that these drugs are better avoided during bridging therapy prior to bispecific antibodies or CAR-T cell therapy. In addition, immunophenotypic changes on blast cells induced by different drugs could also influence risk-based treatment assignment.

## Introduction

Flow cytometry is one of the methods used in the diagnosis of acute lymphoblastic leukaemia (ALL). It provides important information about the expression of antigens on blast cells and is important for determining minimal residual disease (MRD), which allows assessment of response to therapy and further planning of treatment.^1,2^

According to the International Berlin-Frankfurt-Münster Study Group clinical trial (ALL IC BFM 2009) the intensity of therapy for childhood ALL is based on risk stratification.^1^ The patients are divided into three risk groups, standard risk (SR), intermediate risk (IR) and high risk (HR). HR patient must fulfil at least one of the following criteria: presence of specific genetic alterations (t(9;22) or t(4;11)), hypodiploid blast cells, > 1000 blast cells in 1 mL peripheral blood on day 8 of therapy, MRD on day 15 > 10% and patients not in morphological remission on day 33.^1^ SR patients are 1–6 years old, have a white blood cell count of < 20000/mL at the time of diagnosis, < 1000 blasts per mL of peripheral blood on day 8 of therapy, MRD on day 15 < 0.1% and are in morphological remission on day 33 of therapy.^1^ All others are IR patients.^1^

SR and IR patients are treated with 4 consecutive chemotherapy blocks: induction, early intensification, consolidation and reinduction, followed by maintenance therapy. HR patients receive more intensive consolidation with 6 blocks of chemotherapy or 3 blocks followed by bone marrow transplantation.^1^ In induction prednisolone, vincristine, daunorubicin, asparaginase and intrathecal application of methotrexate are used.^1^ In later phases, other cytostatic drugs are administered.^1^

In relapsed or refractory disease, when immunotherapy with bispecific antibodies or chimeric antigen receptor T (CAR-T) cells is commonly indicated, the choice of therapeutic approach is often based on the immunophenotype of the blast cells. For instance the expression of antigens such as CD19, CD20 and CD58 is essential for planning treatment with blinatumomab, rituximab and CAR-T therapy.^2,3,4,5,6^

Some studies have already investigated the impact of the cytotoxic drugs on antigen expression of blast cells and showed that chemotherapy can cause a change in their expression, which may be related to cell death, the efficacy of therapy and the overall prognosis of the disease.^7,8,9,10^ Moreover, chemotherapy may also influence expression of antigens which are essential for effective treatment with bispecific antibodies and CAR-T cells.^10^

Certain antigens expressed on blast cells may also be associated with the prognosis of the ALL patients. Expression of CD27, CD34, CD45 and CD66c antigens has already been reported to be associated with prognosis of ALL.^11,12,13,14,15,16,17,18^ Additionally, expression of CD66c strongly correlates with BCR/ABL rearrangement.^17^ To our knowledge there is no data about the influence of chemotherapeutic drugs on expression of these prognostic markers in the literature. In Table 1 we present the antigens which are important for planning the treatment of ALL patients or are associated with the prognosis of the ALL.

**TABLE 1.** Antigens expressed on acute lymphoblastic leukaemia (ALL) blasts which are essential for planning the treatment or may influence the prognosis of the disease

**Antigen**	**Expression of the antigen in normal and pathological conditions**	**Clinical significance**
CD10	Normal lymphoid progenitors, neutrophils and blast cells of B-ALL^19^	Associated with favourable presenting features in paediatric B-ALL and possible target for emerging CAR-T^19,20,21^
CD19	Normal and malignant B lymphocytes^22^	Target of blinatumomab and CAR-T^22,23,24^
CD20	Normal and malignant B lymphocytes^21^	Target of rituximab, up-modulated in B-ALL patients with poor prognosis^7,21^
CD27	T cells, natural killer cells and thymocytes, also memory B cells, and in some subsets of B-ALL^11^	Positive in B-ALL with BCR/ABL or CRLF2 rearrangement, high positivity was associated with poor prognosis^11,12,13^
CD34	Pluripotent stem cells^14^	Lack of expression associated with worse event free and overall survival of ALL^15^
CD45	Cells of hematopoietic origin^25^	High expression associated with high risk disease and worse event free survival, and worse rate of complete remission after the induction therapy^16,26^
CD58	Hematopoietic and nonhematopoietic cells^27^	Lack of expression reduces the efficacy of blinatumomab and anti-CD19 CAR-T^4,5^
CD66c	Granulocytes and their precursors, most common myeloid antigen on malignant B lymphoblasts^17^	Associated with specific genetic alterations such as BCR/ABL, hypodiploidy, hypodiploidy and CRLF2-positivity in B-ALL^17,18^
CD137	T lymphocytes and natural killer cells but also on activated B cells of naive origin^28^	Enhances B cell survival^28^

B-ALL = B-cell ALL; CAR-T = chimeric antigen receptor T; CRLF2 = cytokine receptor-like factor 2

The aim of the study was to assess the influence of drugs included in ALL induction therapy on antigen expression of blast cells which are common targets for immunotherapy (CD19, CD20) or possess the potential to impact its effectiveness (CD58). Additionally, we have studied the antigens that could play a role in categorizing patients into distinct treatment risk groups, enhancing the precision of patient stratification (CD10, CD27, CD34, CD45, CD66c and CD137). Importantly, we analysed the influence of drugs and their dosage separately.

## Patients and methods

### Patients

Between March 2018 and November 2021, we performed bone marrow aspiration or peripheral blood withdrawal in case of extreme leukocytosis in all children with suspected acute leukaemia. Informed consent was obtained from the patient and parents or consent from a proxy before enrolment in the study. The National Medical Ethics Committee approved the study (protocol number: KME 25/05/15).

First, we performed all routine diagnostic procedures, and if B-cell ALL was confirmed, the remaining bone marrow sample was further cultivated *in vitro*. In total, we recruited 35 patients and collected 34 bone marrow samples and one peripheral blood sample. Six samples were not included in the final analysis because the number of cells after isolation was too low to perform the treatment with cytostatic drugs (4 cases) or because viable cells in the control group were lost during the treatment with cytostatic drugs (2 cases). In total, we treated and analysed blast cells from the remaining 29 samples.

Twenty-seven of the included patients older than one year were treated according to the ALL IC-BMF 09 protocol^1^, more than half of them according to the intermediate risk arm. Two patients younger than 1 year were treated according to the Interfant 06 protocol.^29^ None of the included patients had blasts in the cerebrospinal fluid at the time of diagnosis. One patient who failed induction therapy received anti-CD19 CAR-T cell therapy, followed by bone marrow transplantation. Two other high-risk patients were treated with allogeneic stem cell transplantation. The clinical characteristics and outcome of all included patients are summarised in Table 2.

**TABLE 2.** Clinical characteristics of all included patients

**No. of all included patients with B-ALL**	**N = 29**
Male/female	N
	17/12
Mean age at diagnosis	Years
	4.76 (newborn 17)
Flow cytometric findings at diagnosis	N (%)
Pro-B	3 (10)
Pre-B	6 (21)
Common type	20 (69)
Cytogenetic and molecular finding at diagnosis	N (%)
normal karyotype	6 (21)
hyperdiploid	10 (34)
t(12;21)	(28)
complex karyotype	(3)
MLL	1 (3)
changes of unknown risk potential	3 (10)
Outcome	N (%)
Alive	93)
Died	2 (7)
Cause of death	N
pancreatitis^*^	1
progressive disease^**^	1

* = treated according to ALL IC-BFM 2009 protocol;

** = treated according to Interfant 06 protocol; MLL = mixed-lineage leukaemia rearrangement; N = number of patients; Pro-B, Pre-B and common B = stage of differentiation

The response to therapy in patients treated according to the ALL IC-BFM 09 protocol is summarised in Table 3.

**TABLE 3.** Response to the therapy in patients treated according ALL IC BFM 09 protocol

**Patients treated according to ALL IC-BFM 2009 protocol**	
No. of patients	27
Treatment arm	N (%)
SR	6 (22)
IR	18 (66)
HR	3 (11)
Response to prednisolone on day 8	N (%)
good response^*^	27 (100)
poor response^**^	0 (0)
Minimal residual disease on day 15	N (%)
< 0.1%	10 (37)
0.1–10%	15 (56)
> 10%	2 (7)
Minimal residual disease on day 33	N (%)
neg	16 (59)
< 0.1%	6 (22)
0.1–10%	3 (11)
> 10%	2 (7)
Outcome	N (%)
alive	26 (96)
died	1 (4)

* = prednisolone good response is defined as blast count of less than 1000 per mL of peripheral blood on the day 8 of induction therapy;

** = prednisone poor response is defined as blast count of more than 1000 per mL of peripheral blood on the day 8 of induction therapy; HR = high risk; IR = intermediate risk; N = number of patients; SR = standard risk

Among two patients, who were treated according to Interfant 06 protocol one of them had extreme hyperleukocytosis, finished the therapy and is in a remission. The other patient died of progressive disease.

### Isolation and cultivation of blast cells

After confirming B-ALL diagnosis, blast cells from bone marrow or peripheral blood were isolated by density gradient centrifugation using Ficoll-Paque (GE HealthCare Technologies Inc, Chicago, IL, USA) media and SepMate^TM^-15 centrifugation tubes (STEMCEL Technologies, Vancouver, Canada). After centrifugation and harvesting from the top of the Ficoll layer, cells were washed 2 times in RPMI 1640 medium containing 10 mM HEPES buffer (Gibco, Thermo Fisher Scientific, Waltham, MA, USA). Medium was supplemented with 10% (v/v) fetal bovine serum (FBS, Gibco), Gluta-MAX (100 ×, Gibco), and penicillin-streptomycin (100 ×, Sigma-Aldrich, Merck, Darm-stadt, Germany). Cells were plated at high density in T-75 or T-182 flasks (VWR, Radnor, PA, USA) in cell culture media and incubated in a humidified incubator with 5% CO_2_ at 37°C until the treatment with cytotoxic drugs (maximum 3 days).

### Treatment of blast cells with cytotoxic drugs

Cells were collected and a suspension of 1×10^6^ cells in 1.8 mL of fresh culture medium was plated in each well of the 24-well plate (Corning Inc., Corning, NY, USA). Then, 0.2 mL of different concentrations of cytotoxic drugs (prednisolone, daunorubicin, methotrexate, asparaginase or vincristine) diluted in saline were added to the cells. Each drug was used in three logarithmically increasing concentrations, which were determined before the beginning of the study. Few logarithmically increasing concentrations were tested and final concentrations used in the study were selected based on blast viability. More specifically, the lowest concentration was chosen such that it was not significantly cytotoxic. In the experiments, prednisolone (Predisolut, MIBE GmbH Arzneimittel, Brehna, Germany) was used at final concentrations of 1, 0.1 and 0.01 mM, daunorubicin (Daunoblastin, Pfizer, New York, NY, USA) at concentrations of 1, 0.1 and 0.01 µg/mL, methotrexate (Methotrexate, Medac Pharma, Wedel, Germany) at concentrations of 1, 0.1 and 0.01 mg/mL and asparaginase (Oncaspar Pegaspargase, Servier Pharmaceuticals LLC, Boston, MA; USA) at concentrations of 1, 0.1 and 0.01 units/mL. Vincristine (Vicristine, Teva Pharmaceuticals, Tel Aviv, Israel) was used at concentrations of 0.1, 0.01 and 0.001 µg/mL, with the exception of two experiments, performed at the time when optimal concentration was to be determined. In those two experiments vincristine was used at concentration of 10, 1 and 0,1 µg/mL. Each experiment also included a population of blast cells not exposed to any cytotoxic drug (addition of 0.2 mL saline), which was used as a control. After the addition of the cytotoxic drugs, the cells were incubated in a 5% CO_2_ humidified incubator at 37 °C for three days and then prepared for immunophenotypic analysis by quantitative flow cytometry.

### Quantitative flow cytometric measurements

To quantify the antigen expression a calibration chart was used. It was based on Quantum™ Simply Cellular® beads (Bangs Laboratories Inc., Fishers, IN, USA) and was generated at the time of each experiment. The sample and bead measurements were performed on the same day under the same conditions. The measured fluorescence of each antibody on the surface of the blast cells was converted to an antibody count using the calibration chart and expressed as antibody binding capacity. Measurements, analysis and interpretation of the results were performed according to the Instructions for Use of Quantum™ Simply Cellular® Beads (Bangs Laboratories Inc.).

Cell count and sample preparation for flow-cytometric analysis was carried out as previously described by our group.^30,31^ The sample aliquot containing half a million cells was first put in a test tube. Antibodies against CD10, CD19, CD20, CD27, CD34, CD45 CD58, CD66c and CD137 antigens were added at saturation concentrations (Table 4). 1µL of propidium iodide (concentration 200µg/mL) was also added to determine the viability of cells. After 20 min incubation, erythrocyte lysis was carried out using a commercial lysing solution (BD Biosciences, Franklin Lakes, NJ, USA). The samples were than acquired using a FACSCanto 10-colour flow cytometer (BD Biosciences) with three lasers (405, 488 and 633 nm) and FACSDiva 8.0.2 software (BD Bioscience). The flow cytometer was routinely set up and calibrated by measuring FACSDiva^TM^ CS &T IVD beads (BD Biosciences) and Sphero^TM^ Rainbow Calibration particles (Spherotech Inc., Lake Forest, IL, USA).

**TABLE 4.** The description of the antibodies used for flow cytometry

	**Antibody**	**Antibody clone**	**Antibody vendor**	**Antibody volume (μL)**
CD10	Anti-CD10 Horizon™ BV605	HI10a	BD Biosciences	5
CD19	CD19-BD Horizon™ V450 Mouse Anti-Human CD19	SJ25C1	BD Biosciences	5
CD20	CD20-BD™ CD20 APC	L27	BD Biosciences	7
CD27	CD27-BD Horizon™ APC-R700 Mouse Anti-Human CD27	M-T271	BD Biosciences	5
CD34	CD34-BD™ CD34 PE-Cy™7	8G12	BD Biosciences	5
CD45	CD45-BD™ CD45 APC-Cy™7	2D1	BD Biosciences	3
CD58	CD58-BD Pharmingen™ FITC Mouse Anti-Human CD58	1C3	BD Biosciences	3
CD66c	CD66c-BD OptiBuild™ BV510 Mouse Anti-Human CD66c	B6.2/CD66	BD Biosciences	5
CD137	CD137-BD Pharmingen™ PE Mouse Anti-Human CD137	4B4-1	BD Biosciences	7

**FIGURE 1.**
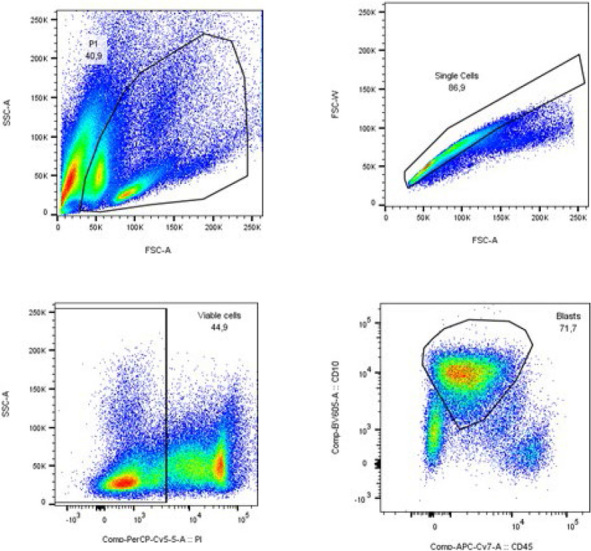
Example of a gating procedure. Mononuclear cells were selected on the forward versus side scatter plot **(A)** and single cells by gating on the area against the width for forward scatter **(B)**. Propidium iodide (PI) was used to select viable cells **(C)**. The final selection of blasts was based on their immunophenotype, in this case by selecting CD10-positive cells on the CD10 versus CD45 plots **(D)**.

For the final analyses, the software BD FlowJo 10.8.1 (BD Biosciences) was used. First a gate for mononuclear cells on the forward versus side scatter plot was selected. In the next step, single cells were selected by gating on the height against the area for forward scatter. Gating on propidium iodide was used to select only viable cells. In the final step, blast cells were gated according to their immunophenotype, in most cases by selecting CD10-positive cells on CD10 versus CD45 plots. An example of a gating procedure is shown in Figure 1. Finally, we determined the fluorescence of each antigen analysed from the histogram.

### Statistical analysis

The analysis was performed using R (R version 4.1.0)^32^ and Julia (version 19.2).^33^ Linear mixed models were fitted using R package nlme (version 3.1–157)^34^ and using MixedModels.jl (version 4.13.1)^35^ in Julia. The optimal transformation of the dependent variables and the assessment of the goodness of fit of linear mixed models were performed with the help of the goodness of fit procedure described in publication by Peterlin *et al*.^36^ The significance of the parameters was assessed with Wald’s test in the nlme package, and the confidence intervals were computed with the help of the parametric bootstrap with 10^6^ repetitions in MixedModels.jl. All p values and confidence intervals are two-sided and were adjusted with the Bonferonni-Holm procedure, with a significance level of 0.05. The marginal effects were calculated with the help of the lmeresampler package (version 0.2.4) with residual bootstrap with 10^4^ repetitions. The 95% confidence intervals for these marginal effects are unadjusted and provided only in graphic form, serving as a tool to aid in understanding the results rather than as definitive statistical result.

We have analyzed every single antigen expression (CDn) with a separate linear mixed model. Before analyzing the data with linear mixed models, we transformed the dependent variables CDn and the number of cytotoxic drugs given. We have done this to improve the resulting models’ goodness of fit. We have used 9 models described by the formula:

$$\log\left(\mathrm{CDn}+0.1\right)∼1+1_{\mathrm{con}}\;*\;\mathrm{con}+\sum_{d\in \mathrm{drugs}}1_{d}*\left(d+k_{-}d*\log_{10}\left(\mathrm{amount}\right)\right)+(1|id).$$

In the above formula, the term ***CDn*** indicates the antigen expression, ***con*** indicates control, ***drugs*** indicates the cytotoxic drugs that were present in this study, ***id*** determines the patient and ***x* → 1_*x*_** indicates an indicator function (**0** if **0** is not true and **1** if ***x*** is true). The fixed effects parameters of the above model are then ***con*** - base value for units in the data that were not exposed to any cytotoxic drugs, ***d*** and ***k_d*** are the - base value and the slope for the units that were exposed to the cytotoxic drug ***d***. We assessed the significance of the parameters ***d*** and ***k_d***.

## Results

For every drug *d* and marker CDn antigen, we have primarily been focused on the statistical significance of the coefficients *d*, which signifies the effect that we observed that was independent of the amount (for the amounts that we have examined), and the coefficient *k_d*, which describes how the value of the log(CD + 0.1) changes with respect to the log_{10} (amount) of a drug *d*, which signifies the effect that we observed that was dependent on the amount. For a given CDn and drug *d* that appear in the same row in Table 5, one of four things can happen:

**A)** Both coefficients (*d, k_d*) were statistically significantly different from 0. In this case, which we highlight with red in Table 5, we can interpret this as merely giving a drug to a cell culture, significantly changing observed antigen expression on blast cells, and that it also matters in which amount the drug is given to the culture. One such example was CD19 and vincristine.
  In the case of CD19 and vincristine, we can therefore expect that the value of the log(*CD* + 01) will decrease by 0.56 − 0.17 * log_10_ (*amount*) for a given patient and amount in the range examined by our study. Note that the amounts of vincristine examined by our analysis ranged from 0.001 to 10. Hence, depending on the amount, we expect the decrease of log(*CD*19 + 0.1) to be between to −0.56 − 0.17 * log_10_(10) = −0.73 to 0.56 − 0.17 * log_10_(0.001) = −0.05 and it decreases linearly with respect to log_10_(*amount*). Other combinations of drug and observed CDn antigen with the same pattern of influence are daunorubicin and CD10, CD137, CD19, CD34, CD45 and CD58, as well as vincristine and CD45 and CD58.
**B)** If just the coefficient *d* was significant, which we highlight with blue in Table 5, we can interpret that giving a drug *d* to cell culture significantly changes antigen expression. However, the amount of drug *d* that is used, if the amount is within the range of the drug *d* given in this study, does not significantly alter the expression of observed CDn antigen. One such example is CD19 and prednisolone.
  In the case of prednisolone and CD19, we can say that we expect that the patient’s value of log(*CD*19 + 0.1) decreases by 0.61, regardless of the amount of prednisolone, as long as this amount is in the range examined by our study, which was 0.01 and 1.0.
  Other combinations of drug and observed antigen with the same pattern of influence were daunorubicin and CD27, as well as prednisolone and CD10, CD27, CD34 and CD58.
**C)** If only the coefficient *k_d* was significant, which we highlight with green in Table 5, we cannot say that just giving some amount (in the range examined by our study) of drug d to blast cells is either up or downmodulating the CDn antigen expression without specifying the amount of this drug. Example of this is CD20 and daunorubicin.
  In the case of daunorubicin and CD20, we can say that the value of patients’ log(*CD*20 + 0.1) changes by −0.22 * log_10_(*amount*) when the amount is between 0.01 and 1, which is the range examined by our study. This means that the value of log(*CD*20 + 0.1) can either change by −0.22 * log_10_(1) = 0 or increase by − 0.22 * log_10_ (0.01) = 0.44, depending on the value of log_10_(*amount*), with respect to which, it changes linearly.
**D)** If neither the coefficient *d* nor *k_d* is statistically significantly different from 0, we cannot say that the drug d affects the CDn marker—an example is vincristine and CD10 (Table 5).
  Marginal effects for these nine models are shown in Figure 2A and Figure 2B. From these figures, we can easily see which cytotoxic drugs change a specific CDn antigen expression. However, since this study is primarily concerned with the effects of cytotoxic drugs conditionally on the individual and these figures are primarily informative, the confidence intervals in these two figures are not adjusted.

**FIGURE 2A.**
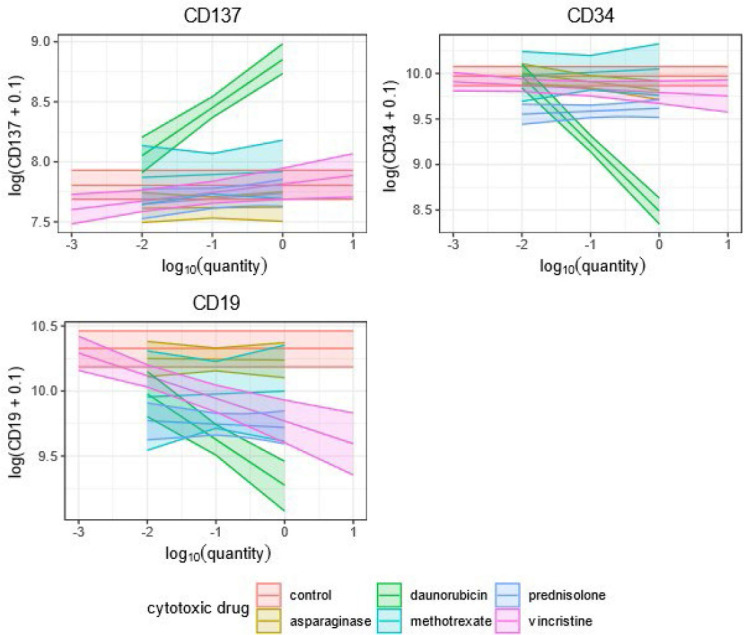
The plots of the marginal effects for the fixed effects coefficients of the models for CD137, CD34 and CD19. The lines correspond to the estimates of the ma rginal ef fects and the coloured areas to the 95% confidence intervals.

**FIGURE 2B.**
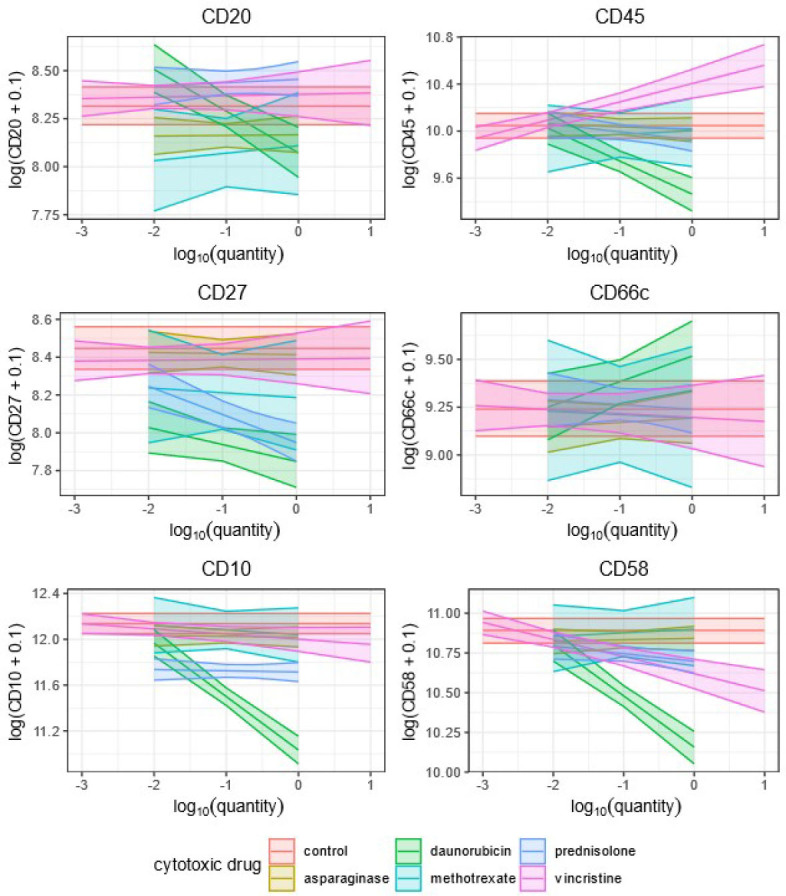
The plots of the marginal effects for the fixed effects coefficients of the models for CD20, CD45, CD27, CD66c, CD10, and CD58. The lines correspond to the estimates of the marginal effects and the coloured areas to the 95% confidence intervals.

**TABLE 5.** The coefficients obtained by using separate mixed model which showed the effect of single drug in CDn antigen expression. The coefficient d showed the presence of CDn antigen and coefficient of k_d the influence of drug dose on antigen expression. The way how the given drug influences the antigen expression is shown in different colours: **RED** (coefficients d and k_d are statistically significantly different from 0), **BLUE** (the coefficient d is significantly different from 0), **GREEN** (coefficient k_d is significantly different from 0) and **WHITE** (neither the coefficient d nor k_d is statistically significantly different from 0). 95% confidence intervals, and corresponding p values are also shown. All confidence intervals and p values were adjusted with Holm’s method

**Drug**	**CD**	**Coefficient *d* (presence)**	**p value for *d***	**Coefficient *k_d* (log(amount))**	**p value for *k_d***
**Asparaginase**	CD10	−0.12, [−0.33, 0.1]	1	−0.01, [−0.11, 0.1]	1
	CD137	−0.18, [−0.46, 0.1]	1	0, [−0.11, 0.12]	1
	CD19	−0.09, [−0.42, 0.24]	1	−0.01, [−0.14, 0.13]	1
	CD20	−0.15, [−0.39, 0.09]	1	0, [−0.09, 0.1]	1
	CD27	−0.03, [−0.28, 0.22]	1	−0.01, [−0.12, 0.11]	1
	CD34	−0.15, [−0.4, 0.09]	1	−0.09, [−0.23, 0.04]	1
	CD45	−0.04, [−0.27, 0.2]	1	−0.02, [−0.16, 0.11]	1
	CD58	−0.05, [−0.23, 0.13]	1	0.01, [−0.08, 0.1]	1
	CD66c	−0.05, [−0.36, 0.27]	1	0.02, [−0.15, 0.19]	1
**Daunorubicin**	CD10	−1.1, [−1.38, −0.83]	< 0.001	−0.47, [−0.63, −0.31]	< 0.001
	CD137	1.05, [0.72, 1.37]	< 0.001	0.4, [0.21, 0.59]	< 0.001
	CD19	−1.05, [−1.48, −0.62]	< 0.001	−0.35, [−0.6, −0.1]	< 0.001
	CD20	−0.24, [−0.53, 0.05]	0.35	−0.22, [−0.39, −0.05]	0.002
	CD27	−0.6, [−0.92, −0.27]	< 0.001	−0.09, [−0.27, 0.09]	1
	CD34	−1.48, [−1.8, −1.16]	< 0.001	−0.74, [−0.93, −0.55]	< 0.001
	CD45	−0.58, [−0.9, −0.27]	< 0.001	−0.28, [−0.47, −0.1]	< 0.001
	CD58	−0.73, [−0.97, −0.5]	< 0.001	−0.32, [−0.46, −0.19]	< 0.001
	CD66c	0.28, [−0.13, 0.69]	1	0.13, [−0.1, 0.37]	1
**Methotrexate**	CD10	−0.1, [−0.52, 0.33]	1	−0.04, [−0.32, 0.24]	1
	CD137	0.11, [−0.33, 0.56]	1	0.02, [−0.24, 0.29]	1
	CD19	−0.33, [−1, 0.35]	1	0.02, [−0.38, 0.42]	1
	CD20	−0.21, [−0.68, 0.27]	1	0.04, [−0.26, 0.34]	1
	CD27	−0.26, [−0.79, 0.27]	1	−0.03, [−0.34, 0.29]	1
	CD34	0.08, [−0.4, 0.56]	1	0.04, [−0.28, 0.35]	1
	CD45	−0.04, [−0.5, 0.41]	1	0.03, [−0.29, 0.35]	1
	CD58	0.01, [−0.25, 0.27]	1	0.02, [−0.21, 0.26]	1
	CD66c	−0.05, [−0.64, 0.54]	1	−0.02, [−0.4, 0.37]	1
**Prednisolone**	CD10	−0.42, [−0.64, −0.21]	< 0.001	−0.01, [−0.12, 0.1]	1
	CD137	−0.06, [−0.32, 0.19]	1	0.05, [−0.1, 0.19]	1
	CD19	−0.61, [−0.94, −0.27]	< 0.001	−0.03, [−0.2, 0.15]	1
	CD20	0.14, [−0.09, 0.37]	1	0.02, [−0.1, 0.14]	1
	CD27	−0.5, [−0.76, −0.23]	< 0.001	−0.15, [−0.3, 0]	0.055
	CD34	−0.35, [−0.6, −0.11]	< 0.001	0.03, [−0.1, 0.16]	1
	CD45	−0.12, [−0.36, 0.12]	1	−0.07, [−0.2, 0.07]	1
	CD58	−0.19, [−0.38, −0.01]	0.034	−0.05, [−0.15, 0.05]	1
	CD66c	0, [−0.19, 0.19]	1	−0.02, [−0.2, 0.15]	1
**Vincristine**	CD10	−0.14, [−0.37, 0.1]	1	−0.05, [−0.14, 0.05]	1
	CD137	0.01, [−0.22, 0.25]	1	0.07, [−0.04, 0.18]	1
	CD19	−0.56, [−0.94, 0]	< 0.001	−0.17, [−0.32, −0.03]	0.004
	CD20	0.06, [−0.19, 0.31]	1	0.01, [−0.08, 0.09]	1
	CD27	−0.06, [−0.34, 0.23]	1	0, [−0.09, 0.09]	1
	CD34	−0.18, [−0.45, 0.1]	1	−0.04, [−0.14, 0.06]	1
	CD45	0.36, [0.07, 0.64]	0.002	0.16, [0.05, 0.26]	< 0.001
	D58	−0.27, [−0.48, −0.06]	0.001	−0.11, [−0.19, −0.03]	< 0.001
	CD66c	−0.04, [−0.39, 0.3]	1	−0.02, [−0.15, 0.11]	1

## Discussion

Our *in vitro* study has shown that prednisolone, methotrexate, vincristine, daunorubicin and asparaginase used in the induction treatment of ALL cause up- or down-modulation of some antigens on ALL blast cells. The effect of the drugs was dose-dependent or dose-independent. We showed that the drugs used in induction therapy influence antigen expression which has been not yet described in the literature such as CD27, CD45, CD137 and CD66c. Moreover, the results of our study showed that cytotoxic drugs used in the induction treatment of ALL can influence CD19, CD20 and CD58 antigen expression on blast cells that can play an essential role in the treatment of resistant or relapsed disease or antigens that could potentially influence the future stratification of patients into risk groups important for planning the treatment.

CD20 expression was stable with all cytotoxic drugs except daunorubicin. When the effect of daunorubicin was analysed with our statistical model, there were no changes in CD20 expression. However, when only the role of dose was examined, a modulation of CD20 expression was observed. Accordingly, we cannot say that just giving some amount of daunorubicin to blast cells causes an up- or down-modulation of CD20 expression without specifying the amount of the drug. These results are difficult to interpret, but as our study was not designed to explain the isolated effect of the dose when there is no effect of the drug in the model. To clarify the discrepancy between the effect of the drug and its dose, further analyses with a larger number of patients and a larger number of different concentrations of daunorubicin are needed.

Dworzak *et al*. have published that CD20 is up-modulated in the early phases of therapy, especially in patients with poor prognosis and high MRD at the end of induction treatment.^7^ Similar changes also occurred after blasts were exposed to different cytotoxic drugs *in vitro*.^7^ Prednisolone and a single intrathecal dose of methotrexate are usually the only drugs used in the first week of ALL therapy and in some protocols, the response to prednisolone is one of the criteria for stratifying patients into risk groups. It is defined by the number of blast cells in the peripheral blood on day 8 of treatment. If the patient has more than 1000 blasts per millilitre of blood, they are defined as a prednisolone poor responder and thus a high-risk patient.^1^ If the number of blasts is very high at the start of treatment, a patient may not reach the threshold despite a relatively good response to therapy, resulting in a classification in a high-risk group with a more aggressive and toxic treatment. None of our patients was classified as HR due to a poor response to prednisolone and CD20 expression remained stable in the *in vitro* settings after the addition of prednisolone. Those results are consistent with the publication by Dworzak *et al*. showing that CD20 up-modulation in early treatment phases is associated with a worse prognosis.^7^ However, since all patients in our experiment responded well to prednisolone, we cannot confirm that CD20 modulation could be a stratification criterion replacing the number of blasts in peripheral blood on day 8 of therapy. To confirm this hypothesis, we would need to include more patients.

Rituximab, an anti-CD20 antibody, has been shown to improve survival in adult patients with CD20-positive B-cell ALL.^2^ Based on the data published by van der Sluijs-Gelling *et al*. describing CD20 up-modulation after *in vitro* exposure of blast cells to prednisolone, dexamethasone, vincristine or asparaginase^10^, one might assume that rituximab has the best efficacy when one of these drugs is used prior to initiation of the therapy. In contrast to the published results, our study suggests that the use of rituximab is equally justified in all phases of therapy that include steroids, vincristine, methotrexate or asparaginase.

CD19 expression in ALL is of clinical importance due to the efficacy of anti-CD19 therapies such as blinatumomab or CAR-T cells.^23,24^ According to our results, daunorubicin, vincristine and prednisolone can induce down-modulation of CD19, which is partly consistent with data published by Van der Sluijs-Gelling *et al*. showing that CD19 is down-modulated after exposure to prednisolone, dexamethasone, vincristine or asparaginase.^10^ These changes may have implications for the treatment of children with resistant or relapsed ALL who receive blinatumomab or anti-CD19 CAR-T therapy. In the 4–6 weeks between leukapheresis and CAR-T administration, bridging therapy reduces the disease burden and keeps the patient’s clinical condition stable. After reviewing the patient’s medical history and response to previous treatment, a multidisciplinary team usually selects cytotoxic drugs for bridging therapy. There are no specific recommendations for the choice of therapeutic approach at this crucial stage.^3^ Our results show that daunorubicin, vincristine and prednisolone can induce down-modulation of CD19 on blast cells suggesting these drugs and especially prednisolone, whose effect is dose-independent, should be better avoided in bridging therapy so as not to impair the effectiveness of anti-CD19 immunotherapy.

The presence of CD58 has been shown to be very important for the efficacy of bispecific anti-CD19 antibodies and anti-CD19 CAR-T cell therapy, as the lack of CD58 decreases T-lymphocyte activation and reduces the success of treatment.^4,5^ In our experiment, CD58 was down-modulated when daunorubicin, prednisolone or vincristine was added. The effect of prednisolone was dose-independent, while the dose of daunorubicin and vincristine was also important. Gaipa *et al*. described the down-modulation of CD58 expression on day 33 of treatment in patients with ALL, and these changes were considered to be prognostically significant.^8^ Our results and the data from Gaipa’s study suggest that it may be useful to avoid daunorubicin, prednisolone or vincristine immediately before specific anti-CD19 therapy as well as for bridging therapy to prevent CD58 down-modulation and a possible decrease in treatment efficacy.

Specific changes in CD10 and CD34 expression during ALL therapy is controversial, but it has been shown that CD10 positivity in B-ALL had been associated with disease prognosis.^15^ CD10, also known as the common acute lymphoblastic leukaemia antigen, can be detected in the blast cells of most patients with B-ALL.^15^ The prognostic significance of CD10 expression is associated with several favourable features such as a hyperdiploid karyotype, age less than nine years and a standard risk group.^15,19^ Our study showed down-modulation of CD10 after treatment with daunorubicin or prednisolone, and the effect of prednisolone was dose-independent. This is consistent with published data showing a transient down-modulation of CD10 in the early phase of ALL treatment, with the changes being more pronounced in patients with a good response to prednisolone and a better prognosis of the disease.^8,9,10,37^

CD34, the pluripotent stem cell antigen, plays an important role in stem cell attachment to the extra-cellular matrix and its presence on blast cells has been shown to be a good prognostic factor in adult patients with ALL, who have undergone stem cell transplantation in the first remission.^14^ Its positivity in ALL is also associated with a lower leukocyte count, a favourable karyotype and a better prognosis compared to CD34-negative ALL.^15^ In our experiment, prednisolone and daunorubicin triggered a significant down-modulation of CD34, which again is partly consistent with published data, where down-modulation of CD34 on blast cells after *in vitro* exposure to prednisolone and *in vivo* during the early phase of ALL therapy has been shown, especially in patients with a better prognosis.^9,10,37^ According to our data, methotrexate, vincristine and asparaginase, drugs also used in induction therapy, had no effect on CD34 expression.

In our study CD10 and CD34 were shown to be down-modulated in blast cells after exposure to prednisolone and daunorubicin, which is partly consistent with data published by Gaipa *et al*. showing correlation between a better response to prednisolone and down-modulation of CD10 and CD34.^9^ Since our study included only a small number of patients and all responded well to predniso-lone, our data do not allow us to draw any conclusions about the importance of changes in antigen expression as a prognostic marker. Therefore, a larger number of patients, including prednisolone-poor responders would need to be enrolled in the study to confirm changes in CD10 and CD34 expression as stratification parameters.

In addition to CD10, CD19, CD20, CD34 and CD58, we analysed other antigens whose modulation significance in patients with B-ALL has, to our knowledge, not yet been described. The experiment allowed us to assess shifts in the expression of CD27, CD45, CD66c and CD137.

Lower expression of CD45, an essential modulator of signal transduction pathways in blast cells, is associated with a higher rate of complete remissions at day 29 of B-ALL treatment and high expression of CD45 was associated with unfavourable prognostic factors and worse event free survival of the disease.^10,16,26,38^ In our experiment, CD45 was down-modulated after exposure to increasing concentrations of daunorubicin, and a dose-dependent up-modulation was observed after the addition of vincristine to the blast cells. Since it was shown that CD45 expression is higher in immature pro-B-ALL than in pre-B-ALL we could speculate that vincristine causes modulation of antigen expression towards the immunophenotype of pro-B-ALL.^16^ Additionally, it has been show that increased CD45 expression in blast cells is associated with decreased cell proliferation and treatment resistance but to determine the importance of changes in CD45 expression after exposure to daunorubicin and vincristine, further test would be needed.^16^

CD137 is known to be present in T lymphocytes and natural killer cells.^39^ The antigen is also expressed in human B lymphocytes, particularly on activated B cells.^28^ Despite the low expression of CD137 (data not shown), we were able to detect an up-modulation of the antigen, but only after exposure to daunorubicin. Zhang *et al*. has published that CD137 mediates cell proliferation and that B cell survival was improved by CD137 ligation.^28^ Potentially in case of CD137 up-modulation on blast cells after exposure to daunorubicin, the survival of blast cells could be improved, influencing the response to therapy. To evaluate the clinical impact of CD137 modulation, further analysis would be needed.

CD27, a member of the tumour necrosis factor receptor superfamily, is mainly present on T cells, natural killer cells and thymocytes.^40^ It can also be detected on the surface of memory B cells as well as on high risk B-ALL with Philadelphia chromosome, cytokine receptor-like factor 2 (CRLF2) rearrangement or in B-other ALL with unknown or not classifying genetic aberrations, where it is additional poor prognostic factor.^11,13,41^ In oncology, CD27 is important in induction of cytotoxic T lymphocyte activation and CD27 agonists were proven to induce antitumor immunity.^13,42^ Additionally Vitale *et al*. have shown that anti-CD27 antibodies have also a direct affect against CD27 positive blast cells.^43^ Our study showed a dose-independent down-modulation of CD27 on blast cells after exposure to daunorubicin or prednisolone. Our results suggest that down-modulation of CD27 may attenuate the efficacy of a direct anti-CD27 antibody against blast cells. Since we have shown that CD27 is down-modulated in blast cells, it would be very interesting to investigate the effect of daunorubicin and prednisolone on CD27 expression and activation of cytotoxic T lymphocytes, for which further studies are needed.

Patients diagnosed with Philadelphia chromosome positive ALL are usually treated with a combination of tyrosine kinase inhibitors and standard chemotherapy.^44^ CD66c is the myeloid antigen commonly expressed on malignant B lymphoblasts, has no clear prognostic significance but is associated with important genetic alterations such as the presence of a Philadelphia chromosome, hyperdiploidy, hypodiploidy and CRLF2-positivity in B-ALL.^17,18^ After analysing the effects of chemotherapy, we found that CD66c expression was mostly low but quantifiable and that none of the drugs had an impact on its expression. Importantly, none of the included patients had Philadelphia chromosome positive ALL. Since the expression of CD66c was stable after the exposure to all tested drugs, CD66c could be a good candidate to MRD detection in patients with CD66c positive B-ALL.

## Conclusions

The present *in vitro* study and statistical model allowed us to determine the effects of each drug and its dose separately. We were able to shown that cytotoxic drugs can induce changes in the expression of antigens that play an important role in planning immunotherapy or that are prognostically important. Importantly we have shown that CD19 and CD58 were down-modulated after exposure to daunorubicin, prednisolone or vincristine, suggesting that these drugs are better avoided during bridging therapy prior to CAR-T cell therapy or bispecific antibodies. Furthermore, we have shown that in some cases the dose of the drug, as opposed to the addition of the drug itself, had no effect on antigen expression. An example of this is prednisolone, where all antigen modulations were dose-independent, meaning that even low doses of the drug can induce immunophenotypic changes.

Moreover, according to our results, cytotoxic drugs trigger other changes in the expression of antigens that could be prognostically important. However, further analyses with a larger number of patients are required to determine the clinical significance of these changes.
